# Wide-field imaging in proliferative diabetic retinopathy

**DOI:** 10.1186/s40942-019-0170-2

**Published:** 2019-12-12

**Authors:** T. Y. Alvin Liu, J. Fernando Arevalo

**Affiliations:** Retina Division, Wilmer Eye Institute, Johns Hopkins University, 600 N. Wolfe Street, Maumenee 708, Baltimore, MD 21287 USA

**Keywords:** Ultra-wide-field imaging, Diabetic retinopathy, Proliferative diabetic retinopathy, Laser photocoagulation

## Abstract

**Background:**

Diabetic retinopathy (DR) is one of the leading causes of vision loss worldwide. For decades, 7-field 30-degree fundus imaging has been the gold standard for DR classification. The aim of this review article is to discuss how the advent of ultra-wide-field (UWF) fundus imaging has changed the management of proliferative diabetic retinopathy (PDR).

**Main body:**

Current data suggests that UWF imaging, as compared to conventional Early Treatment Diabetic Retinopathy Study (ETDRS) fields, detects additional and more extensive PDR pathologies. DR lesions, captured by UWF imaging outside of ETDRS fields, likely carry prognostication value.

**Conclusion:**

UWF imaging represents a major advancement in the detection and management of DR. It remains unclear whether, when and how patients, with PDR changes only peripheral to standard ETDRS fields, should be treated. A larger, prospective, randomized clinical trial is also needed to compare the efficacy of UWF image-guided targeted laser photocoagulation with that of conventional panretinal photocoagulation.

## Background

**Fig. 1:**
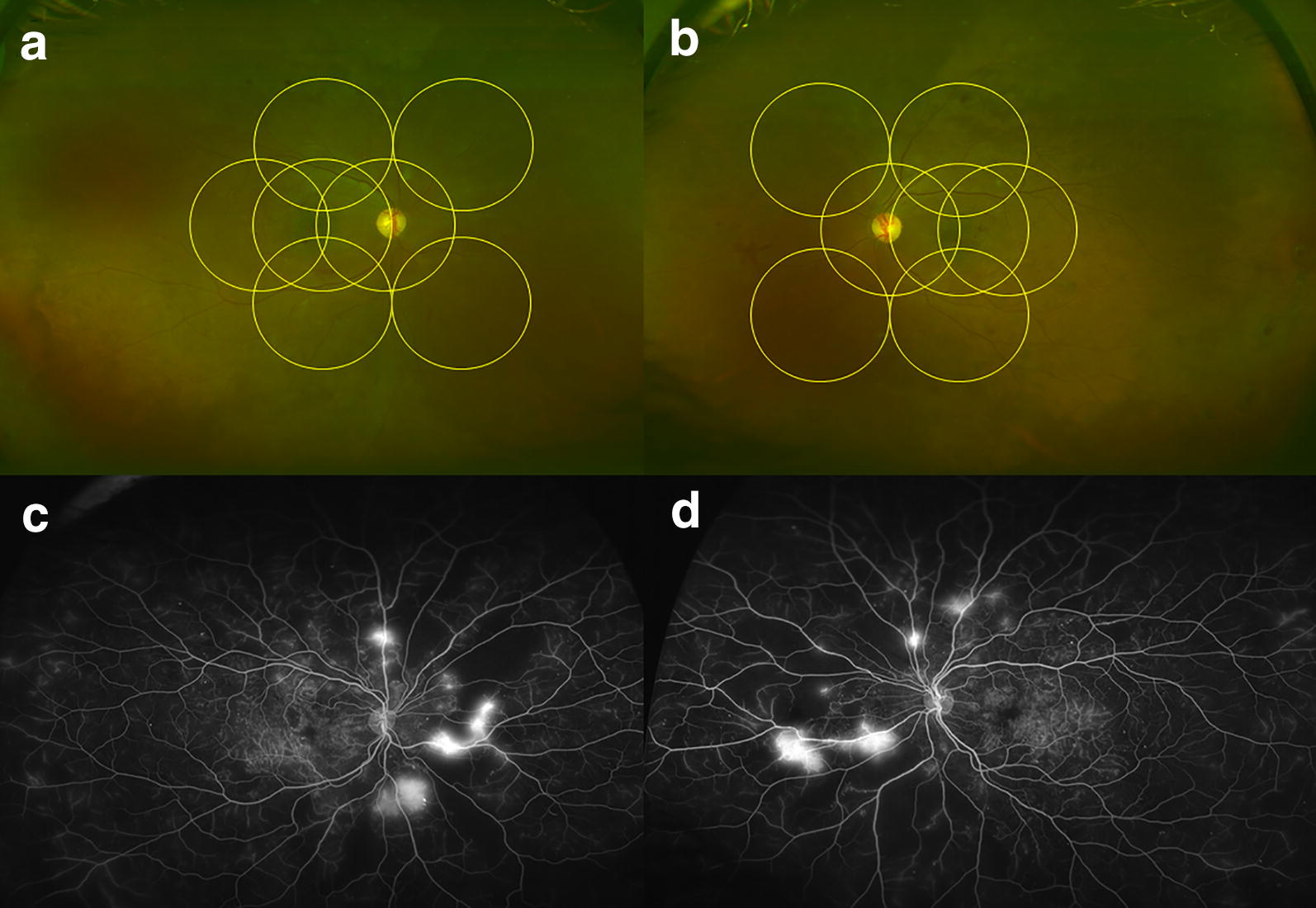
Sample ultra-wide-field imaging of a patient with proliferative diabetic retinopathy in the right (**a**) and left (**b**) eye. Early Treatment Diabetic Retinopathy Study (ETDRS) 7-field, 30-degree fundus images are superimposed in yellow circles. Corresponding fluorescein angiography of the right (**c**) and left (**d**) eye showed leakage from retinal neovascularization and non-perfusion

Diabetic retinopathy (DR) is one of the leading causes of vision loss in the world [1]. With the exponential increase in the incidence of diabetes mellitus, the prevalence and burden of DR is expected to increase dramatically world-wide in the coming decades [2]. Uncontrolled DR can eventually lead to severe vision loss and proliferative diabetic retinopathy (PDR), with features such as neovascularization of iris (NVI), neovascular glaucoma (NVG), neovascularization of disc (NVD), neovascularization elsewhere (NVE), vitreous hemorrhage (VH) and tractional retinal detachment (TRD). DR was first classified in 1968 by a group of experts in Airlie house, Virginia [3]. This classification was subsequently modified for use in the landmark trials of Diabetic Retinopathy Study (DRS) [4] and Early Treatment of Diabetic Retinopathy Study (ETDRS) [5] in the early 1980s and 1990s, respectively. This classification is based on findings within standardized, 7-field, 30-degree fundus photographs of the posterior pole, and has remained the gold standard for decades. In recent years, the advent of ultra-wide-field (UWF) imaging, defined by the Diabetic Retinopathy Clinical Research Network (DRCRnet) as a field-of-view of 100 degrees or more [6], has allowed for visualization of the far peripheral retina, areas that are beyond the field of view of traditional 7-field photographs (Fig. 1). Examples of machines capable of UWF imaging include Optos California^®^ [Optos PLC, Dunfermline, United Kingdom] and Clarus^®^ 500 [Carl Zeiss Meditec, Jena, Germany]. The aim of this review article is to discuss how these newer modalities of fundus imaging has and will change the management of PDR.

## Detection, Prognostication and Treatment

**Fig. 2:**
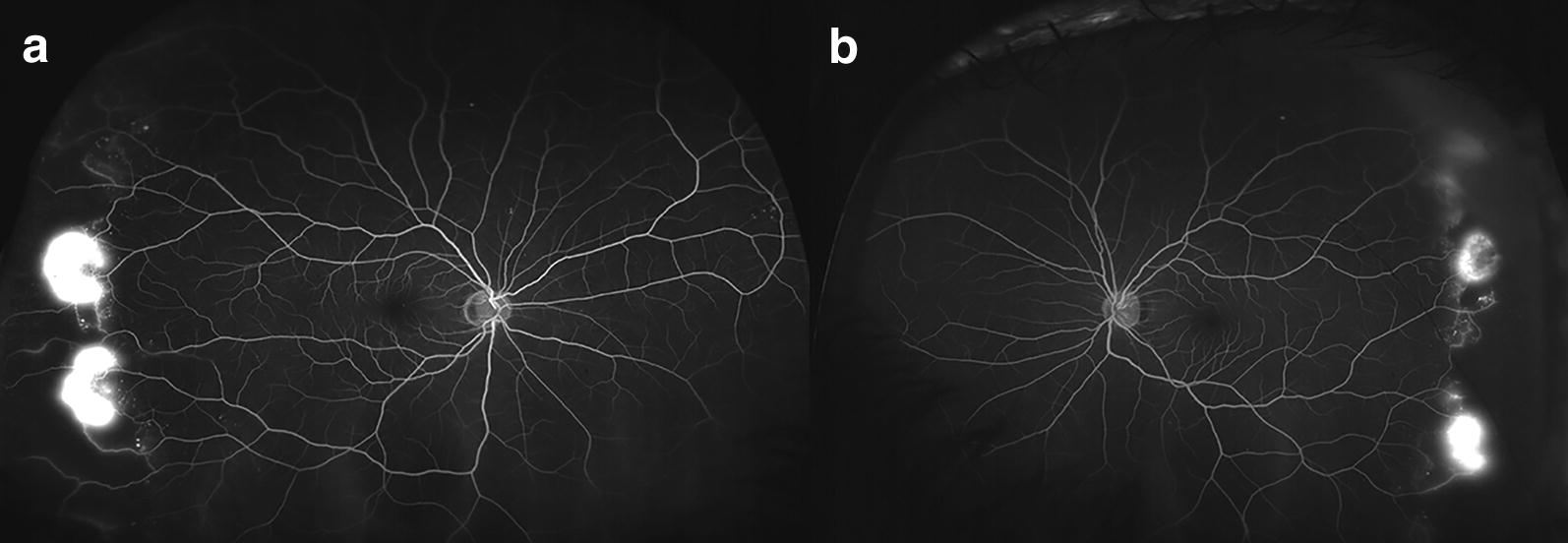
Sample ultra-wide-field fluorescein angiography of the right (**a**) and left (**b**) eye, showing retinal neovascularization outside of the area covered by standard ETDRS 7-field, 30-degree fundus imaging

UWF fundus imaging allows for simultaneous documentation of a wide area of retina and has been shown to be more sensitive in detecting PDR, as compared to standard 7-field, 30-degree fundus imaging. Talks et al. [7]. showed that UWF photographs detected more NVE as compared to both standard DR screening protocol in England (two 45-degree image per eye) and 7-field 30-degree photographs. In 11.7% of the PDR eyes, retinal neovascularization was only detected outside the area covered on 7-field imaging. Wessel et al. [8]. evaluated patients with DR with UWF fluorescein angiography (FA) and superimposed standard 7-fields on these UWF FA images. As compared to standard 7-fields, UWF FA detected 3.9 times more non-perfusion and 1.9 times more NVE, both of which were statistically significant. Similar to findings by Talks et al., Wessel’s study showed that 16.7% of retinal neovascularization were only found outside the area covered on 7-field imaging. An example of PDR with retinal neovascularization only detected outside of ETDRS fields is shown in Fig. 2.

The ability of UWF imaging to capture additional DR features as compared to standard ETDRS fields also provides valuable prognostication information. Silva et al. [9]. showed that the presence of predominantly peripheral lesions (PPL), defined as any DR lesion located predominantly outside of standard ETDRS fields, increased the risk of DR progression and PDR development over 4 years by 3.2 and 4.7 times, respectively. These relationships remained statistically significant, even after adjusting for gender, diabetes type, diabetes duration, hemoglobin A1c levels, and baseline DR severity. In addition, a greater extent of PPLs at baseline also increased the risk of DR progression and PDR development in a statistically significant fashion. Oliver et al. [10]. showed that the presence of peripheral non-perfusion on UWF FA was associated with an increased risk of retinal neovascularization and macular ischemia, and these associations were statistically significantly. However, the caveat was that peripheral non-perfusion was definied as “capillary non-perfusion greater than 1 disc diameter in area outside the vascular arcades,” [10] meaning some of non-perfused areas were likely located within standard ETDRS fields. In addition, Oliver et al. described that the presence of late peripheral vessel leakage, defined as “late venous or arterial hyperfluorescence peripheral to the temporal vascular arcades,” [10] was associated with a statistically significant increase in the risk of retinal neovascularization.

The advent of UWF imaging also led to the practice of UWF-image-guided retinal laser photocoagulation, in which laser treatment was applied selectively to areas of non-perfusion as shown on UWF FA, in contrast to the conventional panretinal photocoagulation (PRP) technique that was first described in the DRS. Various authors [11, 12] have shown that UWF guided laser photocoagulation is effective in treating PDR and leading to regression of retinal neovascularization. Muqit et al. [11]. treated 28 treatment-naïve PDR eyes with targeted retinal photocoagulation. At 12 weeks, 76% of patients showed PDR regression, and 10 out of 28 eyes required repeat treatment. At 24 weeks, UWF FA showed complete disease regression and partial disease regression in 37% and 33% of eyes, respectively. As follow up, Muqit et al. [13]. carried out a pilot randomized trial, which compared targeted retinal photocoagulation with conventional PRP. At 12 weeks, between the UWF-guided targeted retinal photocoagulation group and the standard PRP group, there was no statistical difference in the change in visual acuity and the rate of PDR regression. Specifically, in the targeted retinal photocoagulation group, 60% and 10% of eyes showed partial and complete PDR regression, respectively. In the standard PRP group, 70% and 20% of eyes showed partial and complete PDR regression, respectively. However, the study only involved 30 eyes, and was likely under-powered to detect a difference between the two groups.

There are 2 major limitations to the use of UWF imaging. First, eyelid and eyelash artifacts (see upper left corner of Fig. 1a and lower left corner of Fig. 1b) are frequently encountered. Second, there is image magnification and distortion compared to the original scale, especially in the peripheral aspects of a UWF image where a lesion can be magnified in an exponential manner [14]. Therefore, any UWF imaging study that involves quantification of lesion size and longitudinal evaluation of lesion size changes must take this into account.

## Conclusion and future directions

In summary, UWF imaging represents a major advancement in the detection and management of DR. Specifically, current data suggests that UWF imaging, as compared to conventional ETDRS fields, detects additional and more extensive PDR pathologies. DR lesions, captured by UWF imaging outside of ETDRS fields, likely carry prognostication value in terms of PDR development. These observations will be further evaluated by the DRCR.net Protocol AA, an on-going, prospective, observational, longitudinal study that aims to investigate whether UWF imaging, as compared to standard ETDRS fields, improves our ability to assess DR and predict DR progression over time. It will be interesting to see if the DRCRnet will recommend incorporating peripheral findings on UWF imaging into DR severity grading. In terms of treatment, the natural history of patients, with PDR changes only peripheral to standard ETDRS fields, will need to be elucidated, and it remains unclear whether, when and how these patients should be treated. In addition, a prospective, randomized clinical trial with a larger sample size will be needed to more definitively compare the efficacy of UWF image-guided targeted retinal photocoagulation versus that of conventional PRP.

## Data Availability

Not applicable.

## Abbreviations

DR: diabetic retinopathy
PDR: proliferative diabetic retinopathy
NVI: neovascularization of iris
NVG: neovascular glaucoma
NVD: neovascularization of disc
NVE: neovascularization elsewhere
VH: vitreous hemorrhage
TRD: tractional retinal detachment
DRS: Diabetic Retinopathy Study
ETDRS: Early Treatment Diabetic Retinopathy Study
UWF: ultra wide field
FA: fluorescein angiography
PPL: predominantly peripheral lesion
PRP: panretinal photocoagulation
